# A fast parallelized DBSCAN algorithm based on OpenMp for detection of criminals on streaming services

**DOI:** 10.3389/fdata.2023.1292923

**Published:** 2023-10-31

**Authors:** Lesia Mochurad, Andrii Sydor, Oleh Ratinskiy

**Affiliations:** Department of Artificial Intelligence, Lviv Polytechnic National University, Lviv, Ukraine

**Keywords:** OpenMP technology, clusterization, recommender systems, speed-up, efficiency, silhouette value

## Abstract

**Introduction:**

Streaming services are highly popular today. Millions of people watch live streams or videos and listen to music.

**Methods:**

One of the most popular streaming platforms is Twitch, and data from this type of service can be a good example for applying the parallel DBSCAN algorithm proposed in this paper. Unlike the classical approach to neighbor search, the proposed one avoids redundancy, i.e., the repetition of the same calculations. At the same time, this algorithm is based on the classical DBSCAN method with a full search for all neighbors, parallelization by subtasks, and OpenMP parallel computing technology.

**Results:**

In this work, without reducing the accuracy, we managed to speed up the solution based on the DBSCAN algorithm when analyzing medium-sized data. As a result, the acceleration rate tends to the number of cores of a multicore computer system and the efficiency to one.

**Discussion:**

Before conducting numerical experiments, theoretical estimates of speed-up and efficiency were obtained, and they aligned with the results obtained, confirming their validity. The quality of the performed clustering was verified using the silhouette value. All experiments were conducted using different percentages of medium-sized datasets. The prospects of applying the proposed algorithm can be obtained in various fields such as advertising, marketing, cybersecurity, and sociology. It is worth mentioning that datasets of this kind are often used for detecting fraud on the Internet, making an algorithm capable of considering all neighbors a useful tool for such research.

## 1. Introduction

The clustering of users of online or offline services is frequently used in marketing for producing recommender systems and in cybersecurity for fraud detection (Zhang et al., 2023). Additionally, in the realm of cybersecurity, it's essential to consider various threats, including side-channel attacks. One such side-channel attack worth mentioning is fault attacks, which can compromise the security of clustering algorithms used to detect criminal behavior on streaming services. Frequently clustering of users can be applied to data that is transferred over the network. So to keep data secured it is necessary to apply ciphers to it. As it is stated in the article (Kaur et al., 2022a) one of the options is to use lightweight cryptography, which aims to provide an acceptable level of security at a low cost, particularly in embedded systems with limited resources, such as the Internet of Things devices. One notable cipher in this domain is WAGE, a 259-bit lightweight stream cipher designed for hardware implementation, offering Authenticated Encryption with Associated Data capabilities. However, there are other options to secure future analyzed data. The article (Kermani et al., 2016) discusses error detection approaches for the Camellia block cipher, considering both its linear and non-linear sub-blocks. These approaches can be tailored for different S-box variants, enhancing security and reliability while maintaining acceptable performance. The presented schemes are evaluated through error simulations and ASIC implementations to assess their efficiency. So this solution can highly enhance the security and reliability of our data analysis. As another approach in the article (Aghaie et al., 2017) we found that a lightweight block cipher Midori prioritizes efficient performance and energy consumption, but also this cipher has been enhanced with fault diagnosis schemes to address both malicious and natural faults, improving its reliability. The last option that we thought could be useful to protect our data is the lightweight cryptographic block cipher QARMA which is described in the article (Kaur et al., 2022b). This cipher employs a substitution permutation network (SPN) and error detection schemes, such as cyclic redundancy check, to enhance reliability, with benchmarked performance on FPGA hardware platforms. Using QARMA-64 or QARMA-128 variations ensures data confidentiality and integrity during the transmission of our user's dataset.

Earning a clear understanding of the structural features of a specific group of people and their characteristic features can provide room for better business strategies. Also, such information is a crucial tool for sociological research and can be used by law enforcement agencies for crime control. Therefore, efficient methods for solving this task remain a relevant area of research in machine learning.

One of the most popular clustering algorithms is Density-based spatial clustering of applications with noise (DBSCAN) (Deng, 2020). Among its distinctive features are the absence of assumptions about shapes of clusters and their number, the ability to detect noise, and significantly lower sensitivity to the order of processing elements. These characteristics have made DBSCAN a helpful tool for data analysis in various fields, particularly when dealing with data about people. Such an algorithm is often considered computationally complex. Additionally, clustering a set of user data requires analyzing a large amount of data and conducting various computations. Therefore, there is a need to investigate the efficiency of applying parallel computations (Mochurad and Solomiia, 2020; Mochurad, 2021) to improve this algorithm addressing all the mentioned problems.

This study aims to develop a parallel DBSCAN algorithm variation for processing data of users of a streaming service using parallel computations technique.

The main task of this research is to achieve fast clustering of users of a streaming service by developing a parallel algorithm based on DBSCAN that maintains its distinctive features and demonstrates significant speed-up. As a result, we expect to receive a function from the dataset and additional parameters that will assign each element to a specific cluster by grouping points based on the density of their spatial distribution in the data space.

**Figure 1:**
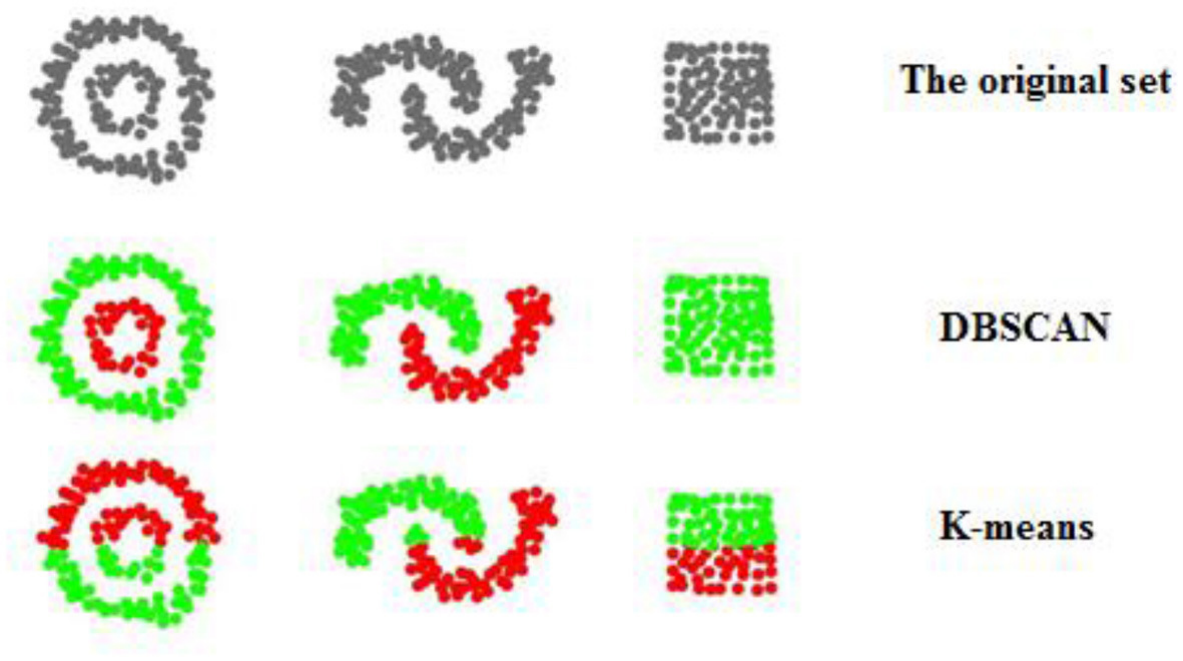
A schematic comparison of the nature of clusters formed by the DBSCAN and K-means algorithms.

As stated before, the main feature of DBSCAN is a consideration of points density (Wang et al., 2020). Comparing examples of the algorithm's performance in a two-dimensional space with the popular clustering algorithm K-means, an enormous difference can be obtained (Figure 1). Each color represents a separate cluster to which a point has been assigned. The original set contains all elements in gray, meaning they are undefined. For each example, the most likely resulting distribution is provided for the corresponding algorithm. Analyzing the clusters, it seems that K-means (Mohiuddin et al., 2020) divides the area rather than the data itself. This is because this algorithm, unlike DBSCAN, does not take into account the relations between neighboring points.

As known (Ester et al., 1996), the computational complexity of the DBSCAN algorithm varies depending on how the nearest neighbors are found. Popular implementations for neighbor search operate data structures such as kd-trees (Shibla and Shibu, 2018), ball-trees (Suchithra and Pai, 2020), RPO-trees (Blelloch et al., 2020), or R-trees (Chen et al., 2010). Such data structures enable faster search of nearby elements in space. By constructing the tree once, time can be saved during the search for neighbor points. Of course, using this approach can lead to a significant improvement in complexity despite the costs of building such trees. However, there are also disadvantages to consider. Firstly, these approaches do not guarantee to find all neighbors, which may be critical in certain cases considering the importance of the number of neighbors for DBSCAN. Secondly, similar data structures may not always meet the expected complexity, as in the worst-case scenario, the complexity of constructing, for example, a kd-tree, can reach the original complexity of *O*(*n*^2^) even in a two-dimensional space. Thirdly, for small to medium-sized datasets, building and using a tree may consume more time than can be saved during the computations.

Certainly, there are various implementations of DBSCAN that use more efficient data structures, but they do not guarantee the same result as the classical algorithm (Hu et al., 2017, 2018; Song and Lee, 2018; Jang and Jiang, 2019; Kim et al., 2019), although they do provide significant speed-up. Additionally, they often require a large amount of data and properly tuned parameters (Wang et al., 2020) to achieve substantial efficiency or may grow with quadratic complexity. Many of these algorithms use sampling techniques that cannot guarantee precise results, although they offer considerable acceleration.

The Dbscan distributed implementation is presented in the work (Wu et al., 2022). The authors exploit hybrid MPI + OpenMP parallelization to take advantage of the resources of modern HPC architectures. This paper presented the Hy-Dbscan algorithm to perform clustering analysis on large-scale scientific data.

Our goal was to accelerate the DBSCAN algorithm without sacrificing its characteristic cluster formation, which directly depends on the correctness of finding neighbors. Therefore, we decided not to use similar data structures or sampling approaches. Such an approach can play a crucial role in clustering data from datasets of various sizes since the number of neighbors found within the eps radius is used to determine the point type in the algorithm, directly affecting the process of merging elements and, consequently, the shape of the resulting subsets. Hence, a predictable and reliable algorithm for finding neighboring points is crucial in this task.

Modern variations of advanced DBSCAN often sacrifice some level of accuracy or are designed only for large datasets. The proposed parallel version of the DBSCAN algorithm maintains the distinctive features of resulting clusters, provides significant speed-up even with medium-sized datasets, and ensures the consideration of all neighbors during its operation, thus achieving high accuracy.

The main contribution of this article can be summarized as follows:

1. A parallel DBSCAN algorithm with a full search for all neighbors was developed, which allowed to avoid redundancy, i.e., the repetition of the same calculations;
2. The choice of the appropriate parallel computing technology is substantiated, which allows to reduce the algorithm's running time in proportion to the number of cores used and to obtain the maximum efficiency;
3. Theoretical performance indicators of the proposed algorithm are calculated, which are confirmed by several numerical experiments;
4. Clustering quality metrics are used to evaluate the accuracy of the result of solving the task.

In this article, we will present the following sections: Section 2 will introduce problem formulation, Section 3 will describe the proposed algorithm, the choice of the appropriate parallel computing technology is justified, and theoretical estimates of the performance indicators of the proposed algorithm are calculated. Section 3 will present the numerical experiments conducted to test the efficacy of the proposed algorithm. Finally, in Section 5, we will conclude our findings and discuss potential avenues for future research.

## 2. Problem formulation

Let's introduce the main notations used in this work: *n*-the number of elements for analysis, *d*-the dimensionality of the data space, *labels*-the resulting sequence of integers of length *n*, *DBSCAN*-the function with four parameters: *data*-a sequence of length *n* containing dataset rows, where the dataset row with index *i* is denoted as *data*[*i*] (hereafter referred to as element or point) and is a sequence of real numbers of length *d*, each corresponding to a dimension of the dataset (the semantic meaning of dimensions is not relevant for the algorithm, so we omit this aspect), *distanceFunction* (hereafter referred to as distance function)–the multidimensional Euclidean distance between two elements in the *data* (1), *eps*-the minimum distance that allows considering two points as neighbors, *minElements*-the minimum number of elements that can be considered as a cluster.


(1) $$\begin{array}{l} distance\;=\;distanceFunction\left(data,\;A,\;B\right)\;. \end{array}$$

where *A* and *B*-the indices of arbitrary points in the dataset, *distance*-a real number that determines the distance between them.

The classical implementation of DBSCAN involves an interface described by Equation (2). The values in the sequence *labels* correspond to the cluster numbers assigned to the points in the dataset. For example, point *i* in the sequence *data* is assigned to the cluster with number stored in *labels*[*i*]. Typically, these are integers starting from −1. All positive numbers represent cluster numbers, while the negative value of one is used to indicate points classified as noise. Such points are considered to be located in the data space in a way that they cannot be assigned to any of the clusters and are considered to be outliers.


(2) $$\begin{array}{l} labels\;=\;DBSCAN\left(data,\;distanceFunction,\;eps,\;minElements\right). \end{array}$$

The task of this research is to achieve fast clustering of users of a streaming service by developing a parallel algorithm based on DBSCAN that maintains its distinctive features and demonstrates significant speed-up on medium-sized datasets. As a result, we expect to receive a function from the dataset and additional parameters that will assign each element to a specific cluster (3) by grouping points based on the density of their spatial distribution in the data space.


(3) $$\begin{array}{l} Y\;=\;DBSCAN\left(X,\;...\right) \end{array}$$

where *X*-input dataset, *Y*-output sequence of labels, *DBSCAN*-the function itself, …–additional parameters.

## 3. Problem solution

### 3.1. Proposed parallel algorithm description

In this work, we propose a parallel version of the DBSCAN algorithm based on the classical method (Ester et al., 1996) with a full search of all neighbors. The idea of our approach lies in the utilization of a reliable parallelization solution, which will save as much time as possible without sacrificing the accuracy of the sequential algorithm.

**Figure 2:**
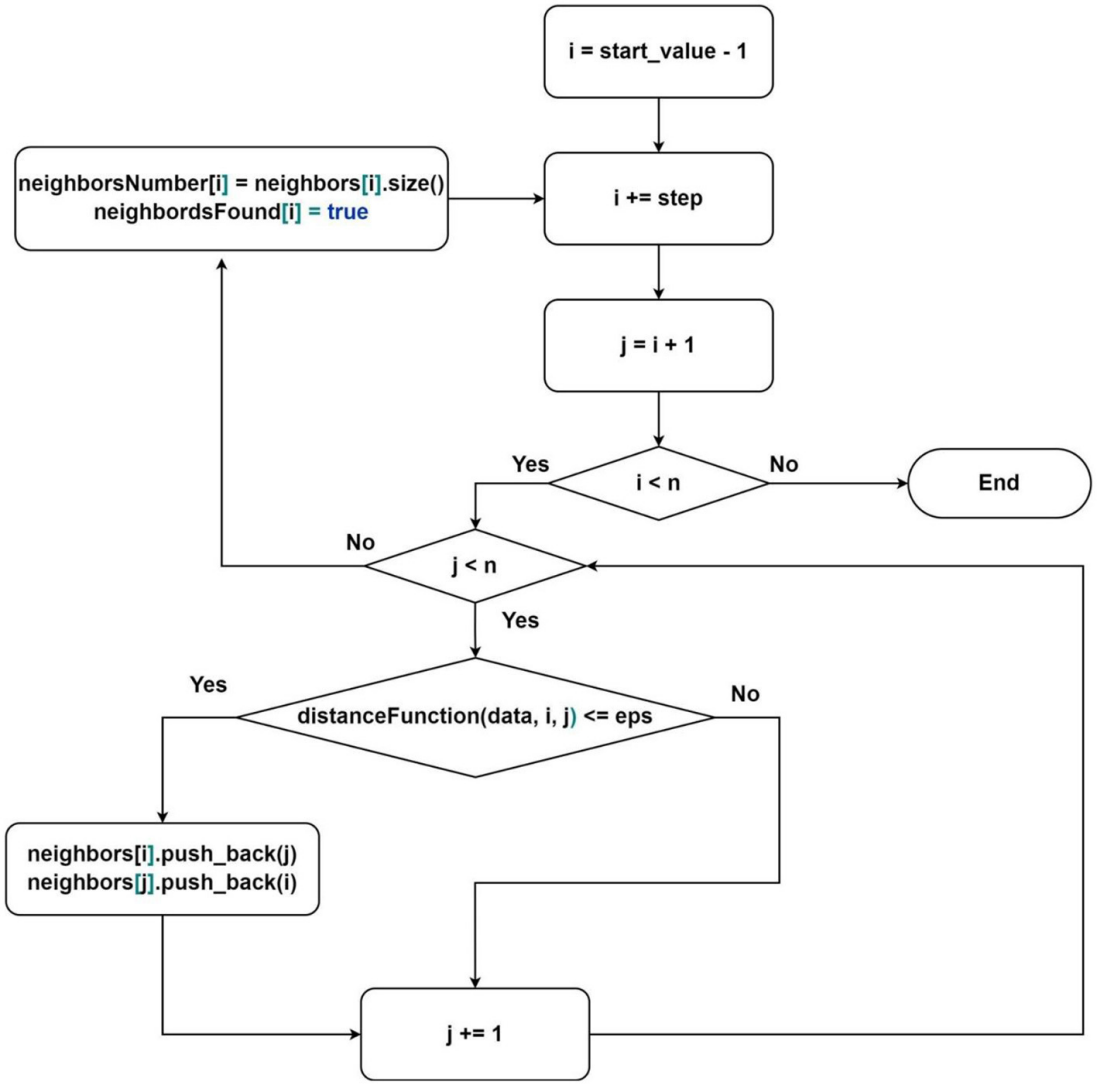
Flowchart of the algorithm for finding neighbors where *neighbors*[*i*], *neighborsNumber*[*i*] and *neighborsFound*[*i*]-sequence of indices of neighbor elements, the number of neighbor elements, and value that represents whether the element was processed for element under index *i*; start_value–the first element that will be processed by current thread, step–elements processing step.

**Figure 3:**
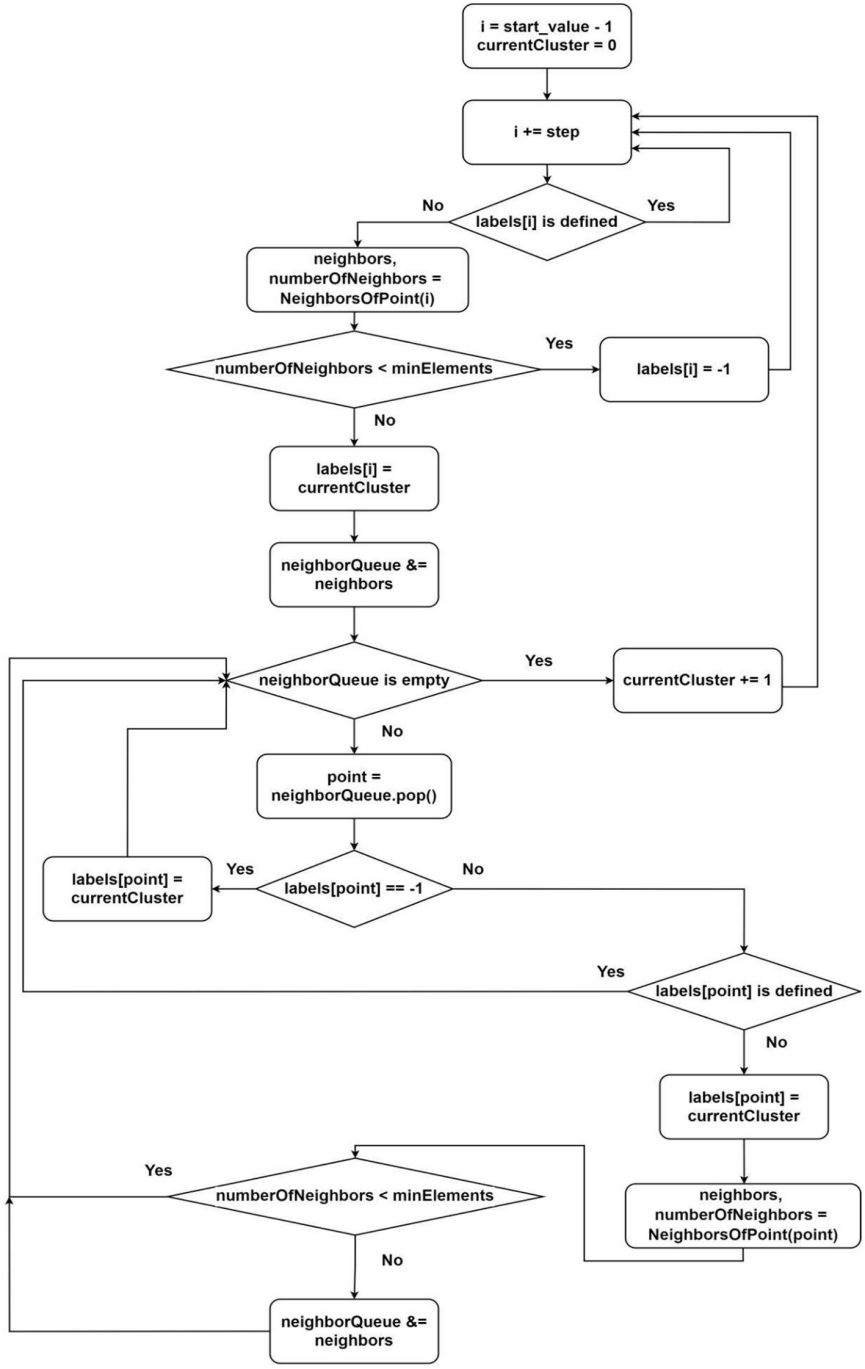
Flowchart of algorithm of assigning the label for point.

Let's divide the algorithm into tasks:

1) Calculation of the distance between points (1);
2) Search for the nearest neighbors (Figure 2);
3) Analysis of a point (Figure 3).

Let's review the algorithm in more detail. The calculation of distances between points should be delegated. We want to work with a hidden implementation of the distance function (1). This approach allows us to choose any method for determining the closeness measure between points based on the specific problem being solved by the user. In this work, we use a specific version of this function which is multi-dimensional Euclidean distance.

The search for neighbors (Figure 2) takes the most time during one iteration of the sequential DBSCAN algorithm since it involves calculating the distances between points in a multi-dimensional space. Processing or analyzing elements (Figure 3) takes slightly less time and requires the result of the neighbors search. Despite this, we consider it beneficial to separate these two tasks, allocating more threads to the neighbors search than to the analysis task. As a result, the waiting time for analyzing threads will tend to zero over time, minimizing delays at the beginning of the algorithm's execution.

Therefore, the proposed algorithm implies the presence of two groups of threads:

1) Group for neighbors search;
2) Group for point analysis.

Let *findNeighboursThreadsNum* be the number of threads for the neighbors search group, and–*defineClustersThreadsNum*be the number of threads for the point analysis group. Then, the total number of threads, denoted by threadsNum, is determined as follows (4).


(4) $$\begin{array}{l} threadsNum=findNeighboursThreadsNum\\ \;+defineClustersThreadsNum \end{array}$$

The first group is responsible for finding subsets of neighbors for each point in the dataset. The neighbors search order is described in Figure 2. It is essential to note that the thread distribution is already provided in this scheme. It is enough to define the parameters *start*_*value* and *step*:


$$\begin{array}{l} start\_value=threadIndex;\\ \;step=findNeighboursThreadsNum; \end{array}$$

Description of the algorithm from Figure 2:

1) Select the next starting point (according to the parameters defined above);
2) For each point in the subsequence:
  - find the distance to the starting point;
  - if the distance is less than or equal to the parameter *eps*:
    - add the index of the current point to the list of neighbors of the starting point;
    - enter the critical section;
    - add the index of the starting point to the neighbors of the current point;
    - exit the critical section;
3) Find the number of neighbors for the starting point and write it to the corresponding memory area in the vector of neighbors counts;
4) Mark the point as processed;
5) Go to the beginning.

The proposed approach of neighbors search allows avoiding redundancy, which occurs when processing points in the classical order. For each next vertex, we need to process one less vertex than in the previous iteration (as seen from Figure 2—*i* > *j*), achieved by processing each pair only once. Additionally, we calculate the number of neighbors for fully processed points to avoid performing this operation multiple times. The marking of processed points is used in the analysis algorithm, which we will consider next.

The second group of threads is responsible for determining the membership of an element in one group or another. During the element processing, we categorize points into core or noise points. The classical algorithm undergoes minimal changes here as it remains efficient (Figure 3). The thread distribution is also provided in this scheme. It is enough to define the parameters *start*_*value* and *step*:


$$\begin{array}{l} start\_value=threadIndex-findNeighboursThreadsNum;\\ \;step=defineClustersThreadsNum; \end{array}$$

This defines the parameters for distributing the iterations of processing the point *i* by the thread with index *threadIndex* (it is guaranteed that only threads with numbers greater than *findNeighboursThreadsNum* will enter this region).

Description of the algorithm from Figure 3:

1) Select the next unclustered point (according to the parameters *start*_*value* and *step*);
2) Request neighbors of the point (there might be waiting since, until the current point's value in the vector of processed points (shared among all threads) becomes true, we cannot guarantee finding all neighbors);
3) If the number of neighbors is less than the minimum required by the algorithm, label this point as noise and go to step 1;
4) Label the point as belonging to the next cluster;
5) For each neighbor, repeat steps 2–5 (for this, we use a queue);
6) Increment the value of the next possible cluster number by one;
7) Go to the beginning.

**Figure 4:**
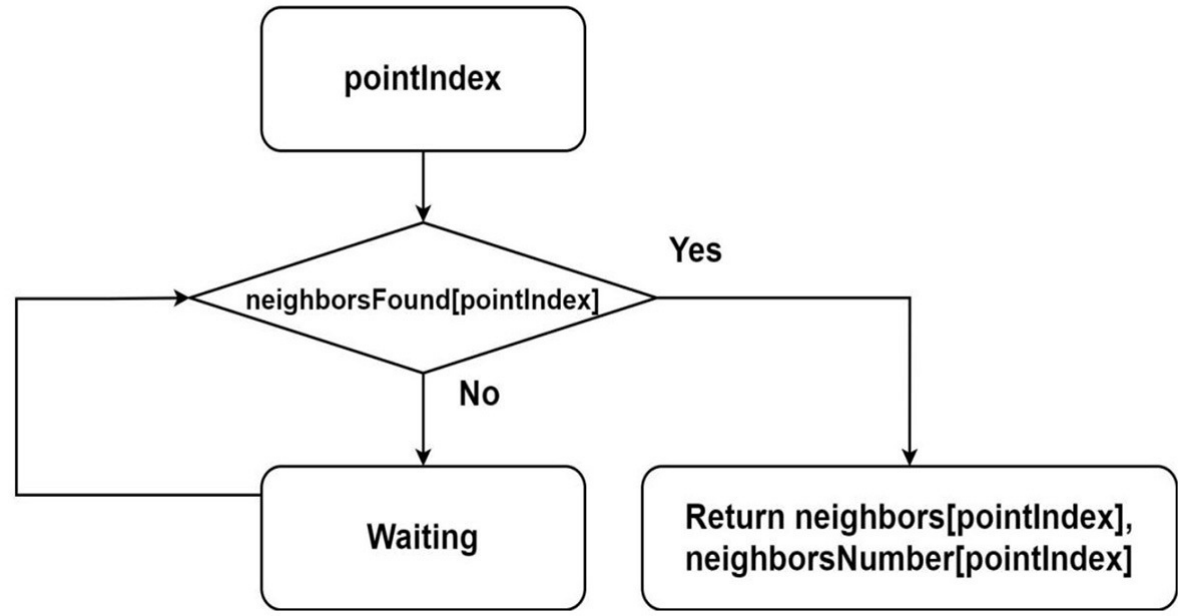
Flowchart describing the algorithm for finding neighbors of a point from parallel threads, where it is assumed that the sequences *neighbors*, *neighborsNumber* and *neighborsFound* are shared among all threads.

A flowchart of the NeighborsOfPoint function is shown in Figure 4. This function is used in the algorithm of assigning the label for point.

### 3.2. Technology selection

Initially, for parallelizing the sequential version of the DBSCAN algorithm was used the threading library of the Python programming language. However, after conducting preliminary experiments and analyzing the results, we noticed that the difference in execution times between the sequential and parallel approaches was practically negligible. This outcome referred to the utilization of the CPython interpreter, which employs a global lock mechanism, allowing only one thread to access shared data structures at any given moment. Consequently, this constraint hindered the effective implementation of true multithreading, resulting in a mere illusion of parallel computation.

As an alternative approach, we attempted to utilize multiprocessing in Python using the multiprocessing library. However, this did not solve our problem either. The main challenge arose from cluster assignment requiring information about neighbors, which is computed in a separate process. This presented challenges in implementing such a memory-sharing format because interprocess memory sharing requires significantly higher resources. Additionally, this could result in excessive time overheads that could not be ignored due to the number of elements in the dataset.

Therefore, we have concluded that we need to utilize technologies where the programmer can fully manage the control over threads. As a result, we conducted an experiment implementing our algorithm using the OpenMP library (Yviquel et al., 2022) in the C++ programming language. This combination of technologies theoretically meets all our requirements, providing a straightforward method to generate parallel threads and exert control over them.

### 3.3. Estimation of computational complexity and expected results

The complexity of the sequential DBSCAN algorithm under worst-case conditions is **O(n**^**2**^**)**, where *n*-the number of elements in the dataset [actually often **O(n**^**2**^**+n)**]. This is partially our case if we do not consider parallel computation, as the work utilizes a complete traversal when searching for neighbors. However, our neighbor-searching algorithm guarantees a complexity always less than **O(n**^**2**^**)** by processing unique pairs of vertices only once. Furthermore, if we appropriately select the number of threads, we expect a uniform distribution with minimal waiting time. Hence, we anticipate a complexity not exceeding $O(\frac{n^{2}+n}{c})$, where *c*-the number of cores.

Thus, the efficiency of our algorithm will approach 1 when the number of threads is appropriately chosen. A proportion for efficiency improvement should be at least 2:1. However, this ratio requires validation through experimentation and depends on various factors, including the dataset's characteristics. The calculation of the expected acceleration of the proposed algorithm will be determined by the Equation (5).


(5) $$\begin{array}{l} S\;=\;\frac{n^{2}+n}{\frac{n^{2}+n}{c}}\sim \frac{n^{2}}{\frac{n^{2}}{c}}\;=\;c, \end{array}$$

where *S*-acceleration, *n*-the number of elements in the dataset and *c*-the number of cores. The expected efficiency of the proposed algorithm will be determined by the Equation (6).


(6) $$\begin{array}{l} E\;=\;\frac{n^{2}+n}{\frac{n^{2}+n}{c}{\;}^{*}\;c}\sim \frac{n^{2}}{\frac{n^{2}}{c}{\;}^{*}\;c}\;=\;1. \end{array}$$

In the research, to verify the correctness of the proposed parallel algorithm, comparisons between the results of sequential and parallel executions were conducted using the Euclidean norm. This approach allows calculating the distance between two points in an n-dimensional space. Initially, the difference between the sequential and parallel results is computed. Then, the Equation for the Euclidean norm is applied to this difference vector. Therefore, if the clustering results for each data sample are identical, meaning the difference vector consists of zeros, the Euclidean norm will also be equal to zero.

In addition, we used the silhouette score described by Equation (7) to evaluate the accuracy of the clustering. In article (Ogbuabor and Ugwoke, 2018), it is mentioned that this score measures how similar a specific data sample is to its cluster compared to others. The silhouette score ranges from−1 to 1. A silhouette score close to 1 indicates that an object fits well into its cluster. In other words, the overall accuracy of the clustering depends on how close the silhouette score is to 1.


(7) $$\underline{\text{S}}\;=\;\frac{\sum_{i=1}^{n}\frac{b\left(i\right)-a(i)}{max(a\left(i\right),\;b(i))}}{n},$$

where *a*(*i*)-the average distance between object “*i*” and all other points within the same cluster; *b*(*i*)-the average distance between object “*i*” and all other points in the nearest neighboring cluster; *max*(*a*(*i*), *b*(*i*))-maximum value between *a*(*i*) and *b*(*i*); *n*-number of objects in the dataset.

In the research, the results' validity was verified using the sklearn library. The function sklearn.cluster.DBSCAN was imported and used to compare the custom parallel implementation of DBSCAN. The clustering results were saved in separate files for different dataset sizes. To assess the reliability of the custom implementation, we calculated the Euclidean norm of the difference between the results from pairs of files with the same dataset size. This comparison provided insights into the validity of the custom implementation compared to the sklearn DBSCAN function results.

## 4. Results

The research task involves applying the proposed algorithm to a normalized dataset containing information about individual Twitch channels of the most prominent streamers in the gaming industry. The objective is to identify objects with similar characteristics and group them into separate clusters for further analysis of the results.

Among the research objectives, the following can be highlighted:

1. Utilizing the proposed parallel DBSCAN algorithm for clustering based on similar feature characteristics of the dataset.
2. Analyzing the results, including both analytical and graphical approaches. In the analytical part, it is necessary to determine the execution times of the sequential and parallel algorithms with different numbers of threads. Parameters such as parallel speedup and efficiency should be computed, along with finding the Euclidean norms of the differences between the results. Additionally, it is essential to investigate the accuracy of clustering and verify its reliability.

We used the “Top Streamers on Twitch (n.d.)” dataset to get further numerical experiment results. This dataset comprises 1,000 rows and 11 columns. Each row provides information about a specific Twitch channel, with features such as Channel, Watch time (Minutes), Stream time (Minutes), Peak viewers, Average viewers, Followers, Followers gained, Views gained, Partnered, Mature, and Language.

Description of features:

1. Channel-Twitch channel name;
2. Watch time (Minutes)-Total viewing time in minutes;
3. Stream time (Minutes)-Total duration of live broadcasts in minutes;
4. Peak viewers-Maximum simultaneous viewers during a live stream;
5. Average viewers-Average viewership during live streams;
6. Followers-Total number of channel subscribers;
7. Followers gained-Number of new followers gained in a specified period (usually a year);
8. Views gained-Total number of views received on the channel's content;
9. Partnered-Boolean value indicating partnership status with Twitch;
10. Mature-Boolean value indicating mature content presence;
11. Language-Language used in the channel's broadcasts.

It is initially necessary to preprocess the input dataset to operate the clustering algorithm effectively. The data needs to be brought into a uniform format, as some features are categorical, and normalization is performed. Therefore, the columns Partnered, Mature, and Language are initially transformed into a numerical form. Subsequently, normalization is applied to the entire investigated dataset, meaning all data is scaled to a single range. Additionally, all Null values and the Channel column are removed, as it is not essential for cluster analysis.

The dimensionality of our normalized dataset has become 1,000 rows by 10 columns, all of which are numerical values. We preserve the obtained dataset in a file, which we will load and utilize in the main program block during experiments.

After executing the algorithm on the normalized dataset, the output consists of an array containing the clustering results. This array has the exact dimensions as the number of rows in the analyzed dataset. Each element of this array corresponds to an object within the dataset. If the value of an array element is −1, the object does not belong to any cluster and is considered noise. Values within the range [0, + ∞) denote the index of the cluster to which the specific object belongs. The resulting array enables us to determine the accuracy of clustering and assess the reliability of the outcomes.

Considering that the dataset consists of 1,000 rows, the study involves examining how well algorithms perform with different amounts of data from this dataset. In other words, we will analyze and compare how long it takes for the algorithms to run on subsets that contain 30, 50, 80, and 100 percent of the dataset.

Two implementations of DBSCAN are compared (see Tables 1, 2):

1) Sequential execution;
2) Parallel execution with a specified number of threads.

**Table 1:** Execution time of the sequential and proposed parallel algorithms with varying thread counts on a 2-core processor, s.

**Percentage of analyzed data, %**	**Sequential execution**	**Threads**		
		**4**	**8**	**10**
30	3.684	2.779	1.969	1.931
50	15.45	12.655	9.072	8.46
80	63.665	52.983	37.93	34.496
100	126.029	99.861	70.609	66.669

**Table 2:** Execution time of the sequential and proposed parallel algorithms with varying thread counts on a 4-core processor, s.

**Percentage of analyzed data, %**	**Sequential execution**	**Threads**		
		**4**	**8**	**10**
30	9.841	5.305	2.936	2.531
50	39.564	20.769	11.622	10.173
80	162.632	95.723	46.733	42.096
100	311.629	178.98	86.351	81.49

The computer configurations used for testing are as follows:

1) AMD Ryzen 5 3500U with Radeon Vega Mobile Gfx, 2,100 MHz, 4 cores, 8 logical processors.
2) Intel (R) Core (TM) i5-6200U CPU, 2,300 MHz, 2 cores, 4 logical processors.

**Figure 5:**
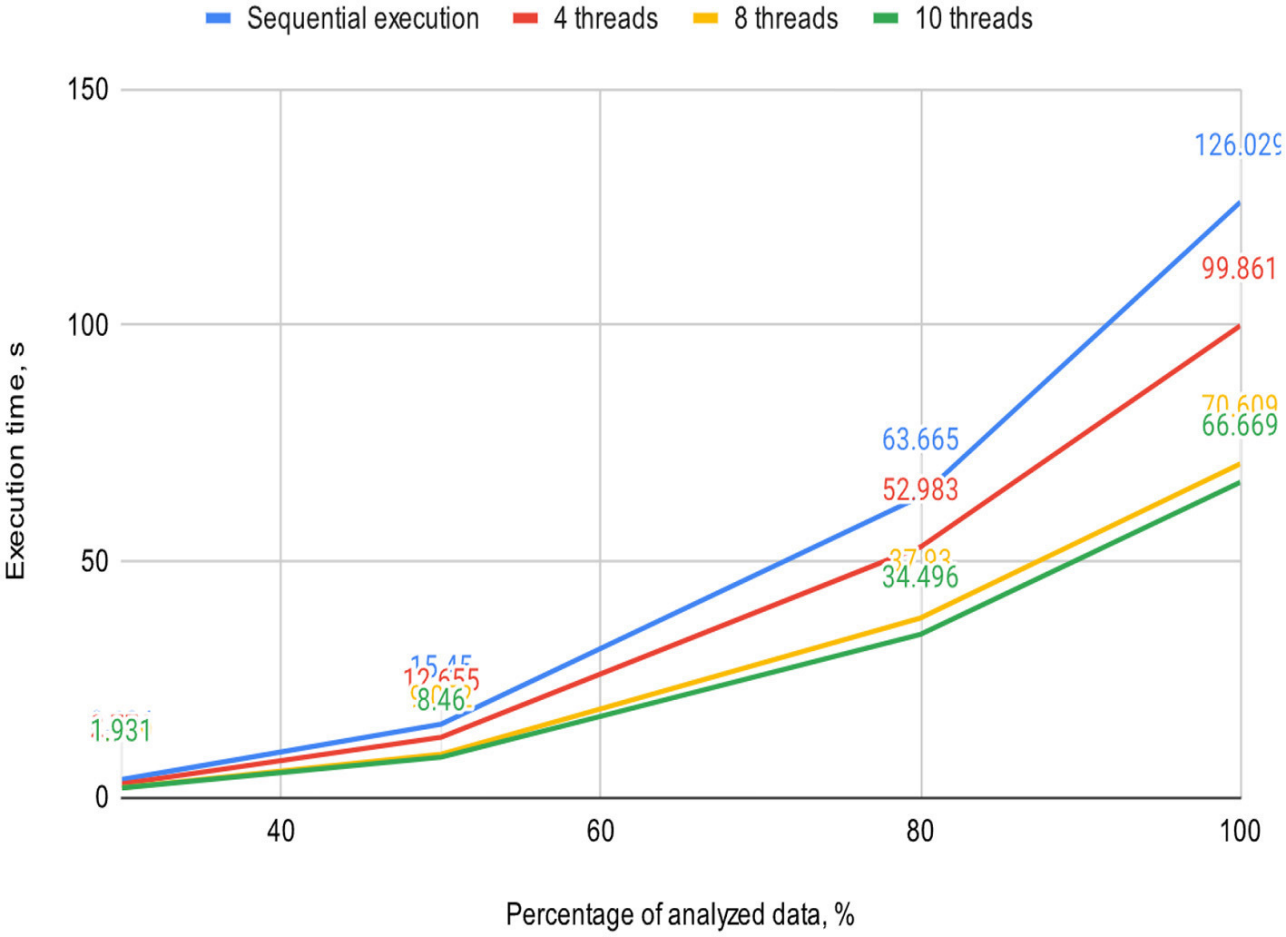
Plot illustrates the execution time dependency of the algorithm on the percentage of analyzed data on a 2-core processor (sequential execution and parallel execution with 4, 8, and 10 threads).

**Figure 6:**
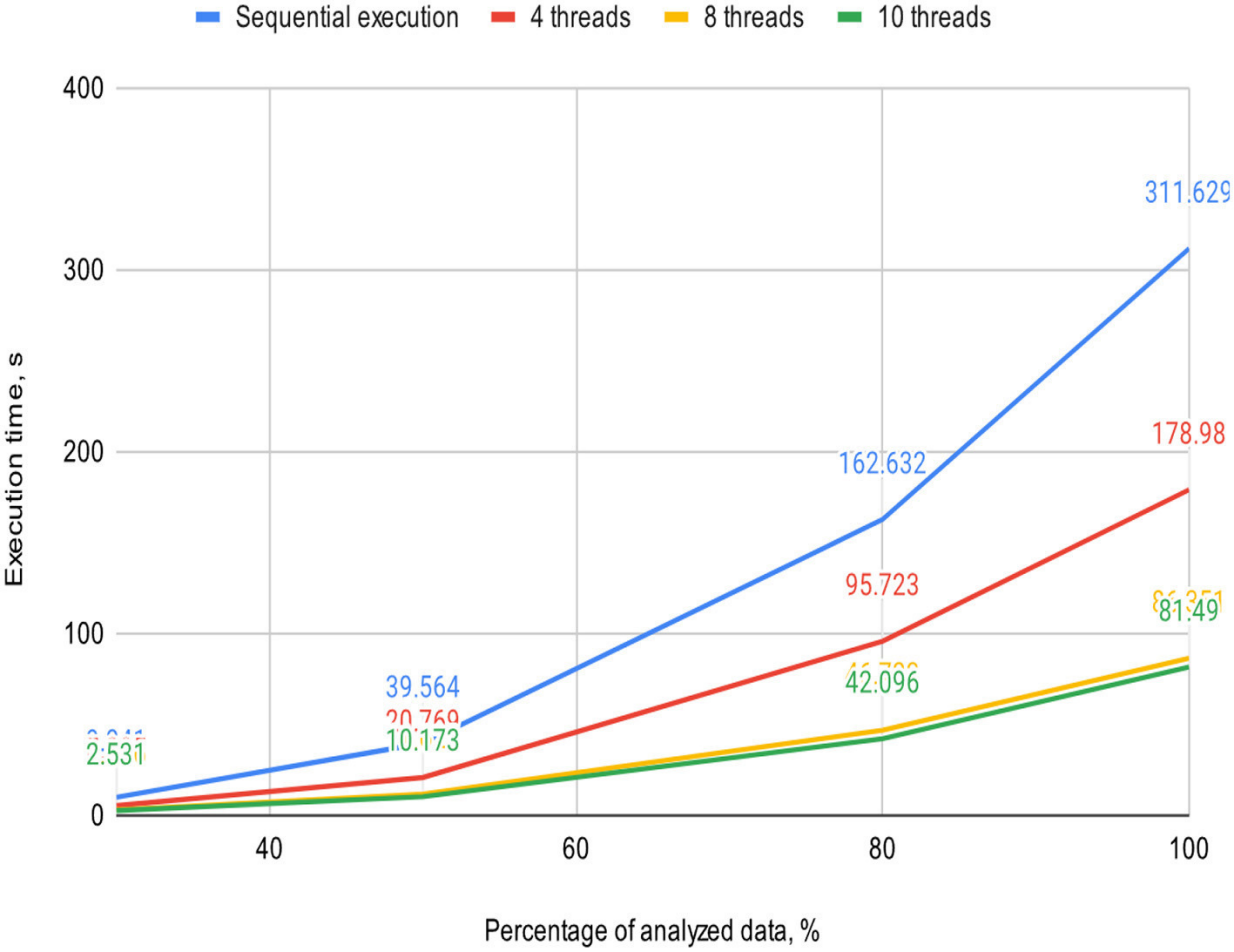
Plot illustrates the execution time dependency of the algorithm on the percentage of analyzed data on a 4-core processor (sequential execution and parallel execution with 4, 8, and 10 threads).

Figures 5, 6 visually represent the results of the proposed algorithm and its comparison with the sequential execution.

**Figure 7:**
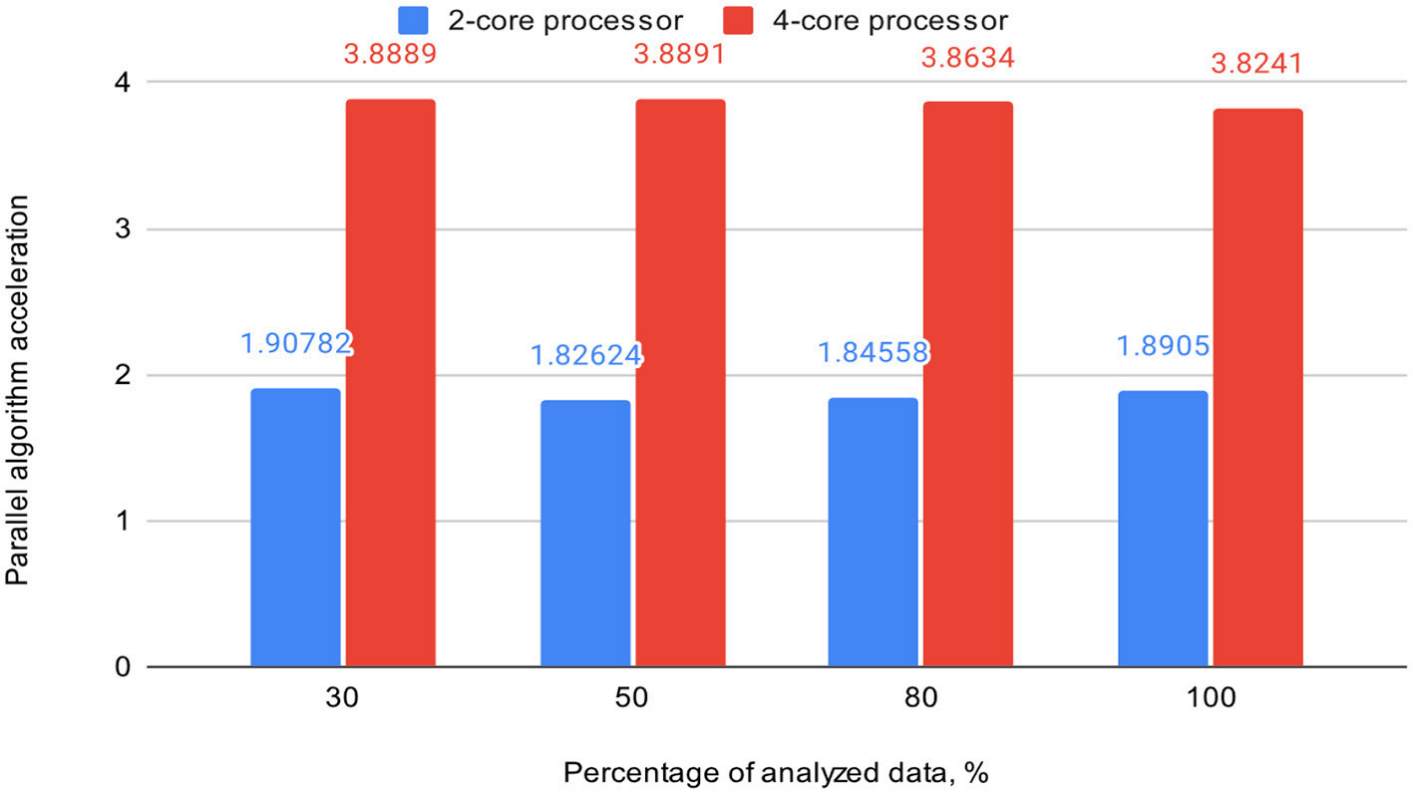
Graph shows the comparison of parallel algorithm acceleration (on 2-core and 4-core processors).

**Figure 8:**
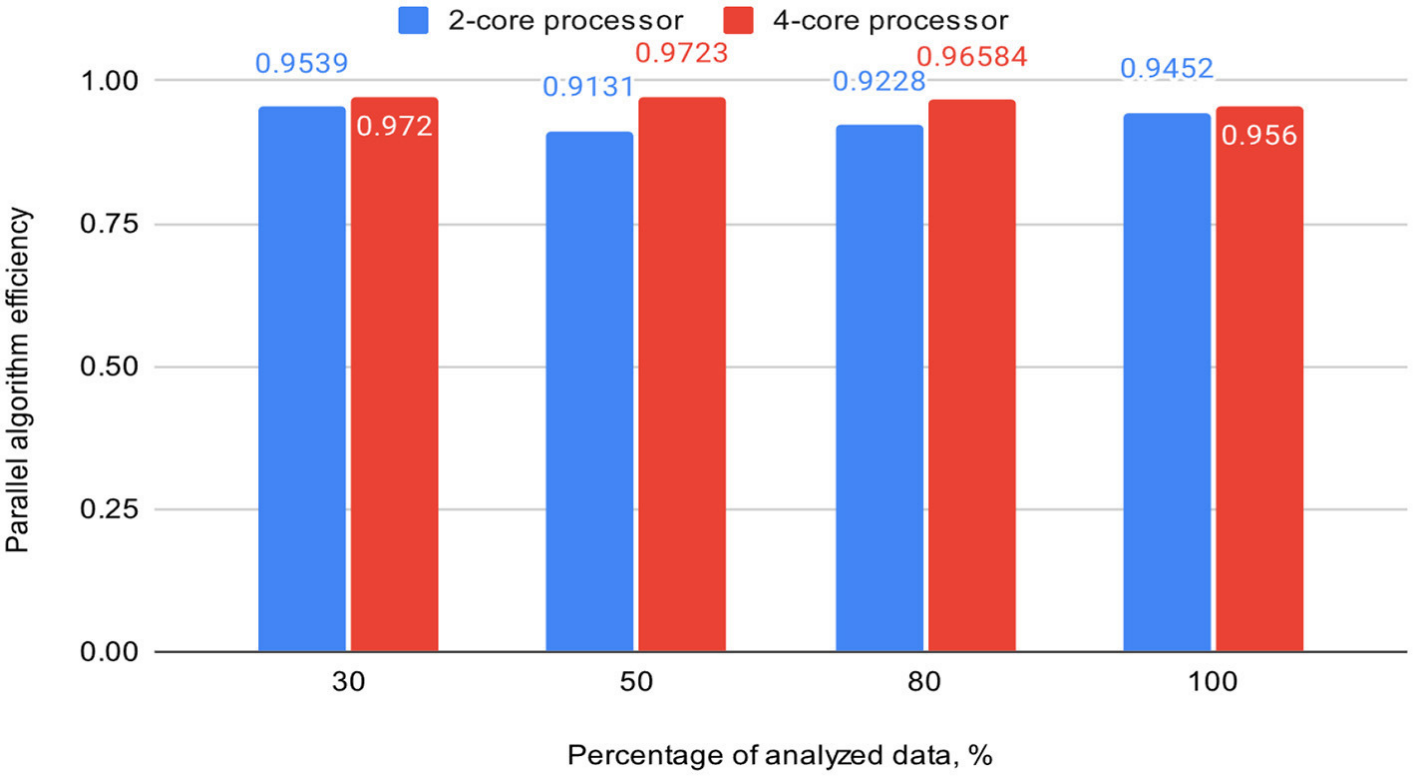
Graph shows the comparison of parallel algorithm efficiency (on 2-core and 4-core processors).

Table 3 also includes acceleration (S) and efficiency (E) metrics for the proposed parallel DBSCAN algorithm on corresponding portions of the investigated dataset. These metrics are visually represented in Figures 7, 8, respectively.

**Table 3:** Acceleration and efficiency metrics of the proposed parallel algorithm (on 2-core and 4-core processors).

**Percentage of analyzed data, %**	**2-core processor**		**4-core processor**	
	**S**	**E**	**S**	**E**
30	1.90782	0.9539	3.8889	0.972
50	1.82624	0.9131	3.8891	0.9723
80	1.84558	0.9228	3.8634	0.96584
100	1.8905	0.9452	3.8241	0.956

Final results were compared with main DBSCAN implementations (see Table 4).

**Table 4:** Execution time of the classical, sequential and proposed parallel algorithms for a certain percentage of data on a 2-core processor, s.

**Percentage of analyzed data, %**	**Classical DBSCAN**	**Sequential DBSCAN (our)**	**Parallel DBSCAN (our)**
30	13.599	3.684	1.931
50	56.612	15.451	8.464
80	265.771	63.665	34.496
100	589.908	126.029	66.669

Moreover, we compared our best results with our classical DBSCAN implementation (contains no additional improvements), our sequential DBSCAN and our best result achieved in this paper. The results of this comparison can be obtained in Table 4. It can be easily seen that our parallel algorithm is way faster than the original DBSCAN. From the table it is possible to make the conclusion that we improved it not only using parallel technologies but also with sequential tools.

It is also important to note that after performing the experiments, all Euclidean norms of differences between sequential and parallel executions across various processor counts are equal to zero. This indicates the complete preservation of the behavioral characteristics of the original algorithm, which was one of our primary requirements. The evaluation of the silhouette score for different data portions to investigate the accuracy of the proposed parallel DBSCAN algorithm and other clustering algorithms is shown in Table 5.

**Table 5:** Silhouette score evaluation for different percentage data quantities from the dataset to investigate the accuracy of the proposed parallel DBSCAN algorithm comparing to other clustering algorithms like K-means, affinity propagation, mean shift, HDBSCAN.

	**Silhouette score**,				
**Percentage of analyzed data, %**	**Our parallel DBSCAN**	**K-means (Mohiuddin et al.**, **2020****)**	**Affinity propagation (Frey and Dueck**, **2007****)**	**Mean shift (Carreira-Perpinan**, **2012****)**	**HDBSCAN (Campello et al.**, **2013****)**
30	0.517	0.535	0.206	0.447	0.172
50	0.562	0.558	0.193	0.436	0.242
80	0.595	0.601	0.191	0.404	0.289
100	0.605	0.601	0.192	0.425	0.322

According to the results stated in Table 5, we can observe that our DBSCAN algorithm managed to conduct high silhouette scores compared to the other clustering algorithms applied to the same dataset. The k-means algorithm provided the best scores at 30 and 80% but lower results than DBSCAN at 50 and 100% of the dataset.

To validate our findings, we computed the Euclidean norms of differences between the outcomes of the sklearn.cluster.DBSCAN function from the sklearn library and our custom implementation across various dataset dimensions. These norms all yielded zero values. Additionally, we calculated silhouette scores for the clustering results using the sklearn.cluster.DBSCAN function. These scores matched those presented in Table 4. Thus, based on the obtained and analyzed results of the proposed parallel DBSCAN, we achieved a significant acceleration of the sequential algorithm without compromising accuracy. Additionally, the earlier theoretical estimates regarding acceleration and efficiency have been confirmed. Table 2 illustrates that acceleration depends on the number of cores, as it was close to 2 on the 2-core processor and close to 4 on the 4-core processor. Furthermore, an indicator that the algorithm has been correctly parallelized is that both on the 2-core and 4-core processors, the parallel efficiency was quite close to 1, as precalculated in the theoretical indicators.

Based on the conducted experiments, a significantly greater number of threads should be dedicated to the neighbor-finding stage rather than the cluster-type determination stage. The lowest parallel execution time and the best parallel efficiency were achieved when utilizing 10 threads, with 8 threads dedicated to neighbor-finding, while two threads were used for cluster type determination. Therefore, the experimentally determined ratio should be ~4:1, though this value is specific to our case. The ratio may vary depending on the dataset size and the subject area under research.

## 5. Conclusions and future works

During our research, we enhanced the classical version of the DBSCAN clustering algorithm. We proposed and implemented parallelization, achieving the expected speedup and efficiency on a dataset of streaming service users. Furthermore, our suggested solution enables significant acceleration, which approaches the number of cores of the computational system, without lowering the accuracy of the result. As the development of multi-core architecture becomes increasingly relevant (Hentosh et al., 2023), the obtained speedup can be significantly improved accordingly. In this work, we also improved the approach to selecting and proportioning the number of parallel threads and task partitioning, enhancing the algorithm's overall performance.

Finally, we achieved a remarkable efficiency of 0.9539 on a dual-core device and 0.9723 on a quad-core one with appropriately chosen thread generation proportions. Our experiments demonstrated substantial speedup when the number of threads for neighbor computation exceeded the number of analyzing threads. We highly recommend utilizing our algorithm based on these findings, as it offers significant performance improvements.

The modification of DBSCAN proposed by us has proven that it is theoretically and practically efficient for clustering datasets of relatively small sizes, particularly concerning user data. However, despite its significant effectiveness, our version of DBSCAN suffers from the drawback of nearly quadratic time increase depending on the volume of data. This drawback arises from the capability of our algorithm to guarantee the discovery of all nearest neighbors, which is one of its advantages. Consequently, this leads us to conclude that further research should be directed toward analyzing methods to scale the algorithm efficiently, seeking ways to obtain better overall processing time for handling massive datasets. By achieving this, the algorithm can become competitive in big data analysis, making it a valuable tool for processing extensive datasets effectively.

In this paper, we presented a parallel modification of classical DBSCAN that is faster and saves original features. Although we demonstrate a way of reaching significant speedup, it is still possible to find methods of improving the results.

We aimed our research at demonstrating the possibility of using parallelism to enhance the DBSCAN algorithm and attempting to integrate such a method as efficiently as possible into the context of the problem. However, at this stage, we consider it advisable to direct future research toward the analysis and comparison of parallel computing tools in order to achieve even better results. The choice of technologies in the field of parallel computing plays an important role, as it affects the percentage of effective use of computer resources. Recently, frameworks that use GPU for achieving better efficiency are becoming more popular due to their ability to perform some operations faster than CPU. Therefore, we consider it is possible for our algorithm to take advantage of modern technologies like CUDA.

Also, a crucial stage of our research was the task of thread distribution. As mentioned in previous sections, to achieve our best results, we had to distribute threads unevenly among tasks. But maybe it is possible to avoid such considerations. Automatic determination or adaptation of threads' distribution can save a reasonable amount of time. That's why we consider it promising to focus future research on studying this issue, for example, using dynamic parallelization methods.

## Data availability statement

The original contributions presented in the study are included in the article/supplementary material, further inquiries can be directed to the corresponding author.

## Author contributions

LM: Conceptualization, Data curation, Formal analysis, Investigation, Methodology, Project administration, Writing–original draft, Writing—review & editing. AS: Resources, Software, Supervision, Visualization, Writing—review & editing. OR: Funding acquisition, Validation, Visualization, Software, Writing—review & editing.
